# Cancer Health Empowerment for Living without Pain (Ca-HELP): study design and rationale for a tailored education and coaching intervention to enhance care of cancer-related pain

**DOI:** 10.1186/1471-2407-9-319

**Published:** 2009-09-09

**Authors:** Richard L Kravitz, Daniel J Tancredi, Richard L Street, Donna Kalauokalani, Tim Grennan, Ted Wun, Christina Slee, Dionne Evans Dean, Linda Lewis, Naomi Saito, Peter Franks

**Affiliations:** 1 Department of Internal Medicine, University of California, Davis, School of Medicine, Sacramento, California, USA; 2 Department of Pediatrics, University of California, Davis, School of Medicine, Sacramento, California, USA; 3 Center for Healthcare Policy and Research, University of California, Davis, Sacramento, California, USA; 4 Department of Anesthesiology and Pain Medicine, University of California, Davis, Sacramento, California, USA; 5 Kaiser Permanente, Northern California, California, USA; 6 UCD Cancer Center, Sacramento, California, USA; 7 Department of Family and Community Medicine, University of California, Davis, School of Medicine, Sacramento, CA, USA; 8 Department of Communications, Texas A & M University, College Station, TX, USA

## Abstract

**Background:**

Cancer-related pain is common and under-treated. This article describes a study designed to test the effectiveness of a theory-driven, patient-centered coaching intervention to improve cancer pain processes and outcomes.

**Methods/Design:**

The Cancer Health Empowerment for Living without Pain (Ca-HELP) Study is an American Cancer Society sponsored randomized trial conducted in Sacramento, California. A total of 265 cancer patients with at least moderate pain severity (Worst Pain Numerical Analog Score >=4 out of 10) or pain-related impairment (Likert score >= 3 out of 5) were randomly assigned to receive tailored education and coaching (TEC) or educationally-enhanced usual care (EUC); 258 received at least one follow-up assessment. The TEC intervention is based on social-cognitive theory and consists of 6 components (assess, correct, teach, prepare, rehearse, portray). Both interventions were delivered over approximately 30 minutes just prior to a scheduled oncology visit. The majority of visits (56%) were audio-recorded for later communication coding. Follow-up data including outcomes related to pain severity and impairment, self-efficacy for pain control and for patient-physician communication, functional status and well-being, and anxiety were collected at 2, 6, and 12 weeks.

**Discussion:**

Building on social cognitive theory and pilot work, this study aims to test the hypothesis that a brief, tailored patient activation intervention will promote better cancer pain care and outcomes. Analyses will focus on the effects of the experimental intervention on pain severity and impairment (primary outcomes); self-efficacy and quality of life (secondary outcomes); and relationships among processes and outcomes of cancer pain care. If this model of coaching by lay health educators proves successful, it could potentially be implemented widely at modest cost.

**Trial Registration:**

[Clinical Trials Identifier: NCT00283166]

## Background

An estimated 90% of patients with cancer experience at least moderate pain at some point in their illness, and 42% of patients receive inadequate palliation [1] Aside from impairing quality of life, uncontrolled pain can contribute to depression, increase the likelihood of suicide, and decrease patient acceptance of potentially beneficial therapy[1] Barriers to effective pain control reside with health care systems, physicians and patients[2,3] While efforts to address system- and provider-level barriers must continue, patients and their families represent an attractive target for interventions because they stand to gain the most from effective pain management and because activated patients have considerable influence on physician behavior[4,5] The Ca-HELP (Cancer Health Empowerment for Living without Pain) Study is a randomized controlled trial of a brief patient activation intervention. This article describes the rationale and conceptual model underlying the study, the Tailored Education and Coaching (TEC) protocol, the design and administration of the study, and the planned analytic approach. Results from the study are expected to be published in 2010.

In a series of articles published in the late 1980's, Greenfield, Kaplan, and colleagues showed that expanding patients' involvement in care can improve patient outcomes[4,6] In the cancer domain, at least six separate, randomized controlled trials have shown that educational interventions with components emphasizing self-care or activation can improve care of cancer-related pain [7-12] All of these studies delivered various combinations of information and support. In general, more intensive interventions involving human interaction and support were associated with larger effects on pain-related processes and outcomes. However, no studies explicitly tested individually tailored interventions to enhance pain-related communication with the treating physician as well as pain self-management. In addition, several practical and theoretical issues have limited the introduction of these interventions into practice: the interventions have frequently required highly specialized personnel, were often applied to highly selected subgroups, made few attempts to measure mediating or moderating variables, were not generalizable to clinically and demographically heterogeneous populations, and generally emphasized patient education over than participation in care.

In a pilot evaluation of the intervention described in this article, Oliver et al randomized English-speaking adults with cancer and moderate pain to a 20-minute TEC session (n = 34) or to a control group (n = 33)[13] At baseline, there were no significant differences between experimental and control groups in terms of average pain, functional impairment due to pain, pain frequency, or pain-related knowledge. However, average pain at follow-up improved significantly more among experimental patients (p = .014). Subsequent work by Kalauokalani et al. showed that benefits of the intervention were concentrated among ethnic minorities, leading to a reduction in health disparities[14]

The current study evaluates a brief, patient-centered, theory-driven intervention that, if shown to be effective, could be widely disseminated. The TEC intervention developed for this study is rooted in Social Cognitive Theory (SCT) as developed by Bandura.[15] This theory posits that behavior change and maintenance are largely a function of expectations about one's ability to engage in or execute the behavior[16] In Bandura's conceptualization, self-efficacy is derived from four sources: previous performance, vicarious experiences, verbal persuasion, and mood. The first three sources impact self-efficacy directly. The TEC intervention creates the expectation of success by simulating successful interactions with the physician through role play, describing the success of other patients, and persuading the patient of the benefits of greater participation in care.

**Figure 1:**
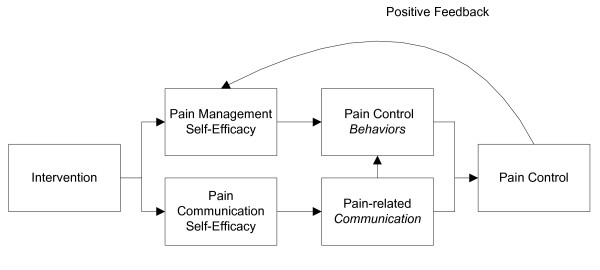
**Ca-HELP Conceptual model**.

Bandura's notion of self-efficacy is task specific. We hypothesized that two forms of self-efficacy may be germane to cancer pain management. *Pain management self-efficacy *is confidence in the ability to achieve control over one's pain. *Communication self-efficacy *is confidence in the ability to communicate effectively about pain with one's physician. These two forms of self-efficacy may mediate pain relief via separate pathways. As depicted in Figure 1, pain management self-efficacy may lead to a greater sense of pain-related mastery and control, which in turn promotes less pain, anxiety, and functional impairment[13] Communication self-efficacy leads theoretically to more assertive interactions with the physician, more effective clinical interventions (e.g., provision of stronger analgesics), and better outcomes.

Effective pain self-management requires the patient to apply a complex set of skills that involve monitoring, medicating (for chronic and breakthrough pain), anticipating (side effects), and communicating with the treating physician. SCT predicts that patients with greater self-efficacy for these behaviors will have a higher likelihood of actually performing them. The theory finds empirical support in a meta-analysis of psychosocial interventions among cancer patients[17] In that study, Graves showed that interventions with more SCT components had larger effect sizes than interventions with fewer SCT components.

This article describes the design and implementation of the Cancer Health Empowerment for Living without Pain (Ca-HELP) Study.

## Methods/Design

### Study design

**Figure 2:**
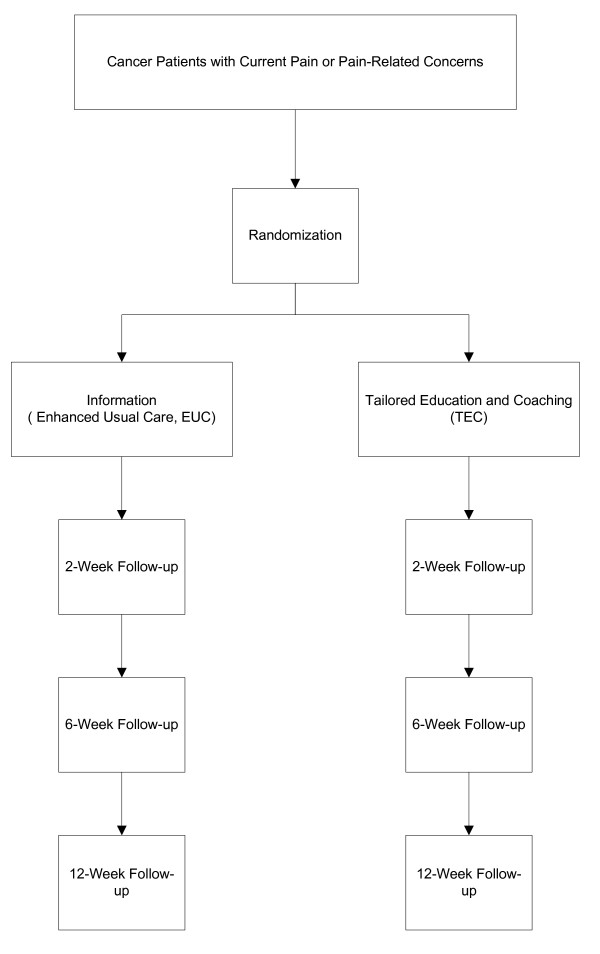
**Study design**.

The study was designed as a randomized controlled trial comparing TEC to educationally-enhanced usual care (EUC). The unit of randomization is the individual patient. The choice of design reflects several considerations. We used EUC rather than usual care as the comparator in order to control for the effects of time, information-giving and companionship. Thus, any improvement in observed pain outcomes would owe to the effects of activation-coaching over and above what might be accomplished by having an empathetic lay person provide educational materials and engage in supportive conversation. We chose to randomize individual patients rather than physicians or systems because: 1) the intervention is posited to exert its effects primarily by affecting patient knowledge, attitudes, self-efficacy, and behaviors; and 2) based on the known difficulties in consistently modifying physician behavior [18,19], spill-over effects (changes in physician behavior as a result of seeing multiple intervention patients) were expected to be minimal. The overall study design is depicted in Figure 2.

### Selection of study sites and physicians

Cancer care physicians were recruited from three health systems (UC Davis Cancer Center; Kaiser-Permanente Sacramento/Roseville; and the VA Northern California Health System) and one private practice, all in Northern California. Medical, radiation, and (after March 2008) gynecological oncologists (including both staff physicians and clinical fellows) were deemed eligible if they saw patients at one of the participating sites and were in clinical practice at least 20% time (i.e., at least 1 full day per week). Physicians gave blanket consent to contact all their eligible patients, or in several instances, selected patients only. Among the 49 participating physicians, 23 (47%) authorized audiorecording of the index study visit. Physicians were offered feedback on the outcomes of their own patients compared with the aggregate results but received no monetary compensation.

### Patient eligibility, recruitment, consent, and randomization

Ethical approval was obtained by the Institutional Review Boards of University of California, Davis and the Kaiser Foundation Research Institute. Patients eligible for enrollment in the study included all cognitively intact, English-speaking adults obtaining care (active treatment or surveillance) from participating oncologists for selected solid tumors and who reported more than minimal cancer-related pain. "Cancer-related pain" was defined as "pain that you would bring to the attention of your cancer doctor" (regardless of the actual etiology). More than minimal pain was defined as a score of 4 or greater (on a scale of 0-10) for "worst pain past two weeks" or pain that during the same period interfered at least moderately with function. Specific inclusion and exclusion criteria are presented in Table 1.

**Table 1:** Patient inclusion and exclusion criteria.

Inclusion Criteria	Exclusion Criteria
Seen or scheduled to be seen at participating facility	Major surgical procedure scheduled within six weeks

Age 18 to 80	Enrolled in hospice

Diagnosis of locally advanced or disseminated lung, breast, prostate, head & neck, esophageal, colorectal, or gynecologic cancers*	Followed by pain management service (more than one visit made or scheduled)

English speaking	Already contacted for study

Recent worst pain (past two weeks) reported to be 4 or higher (on a scale of 0 to 10) OR pain in past two weeks reported to have interfered with normal daily activities at least moderately (at least 3 on a 5-point scale).	Difficulty thinking or expressing himself
	
	Unable to receive and/or complete mailed enrollment materials

*Patients with pancreatic cancer were included initially, but this diagnosis was removed from the list of eligible cancers on June 1, 2007 upon the recommendation of the Data Safety and Monitoring Board.

Potentially eligible patients were identified with the assistance of participating oncologists, who provided computer-generated lists of patients who had been seen within the past 3 months (UCDMC) or were scheduled to be seen within the next two months (Kaiser, VA, and private practice). Personalized letters were prepared notifying patients of the aims of the study and requesting assent to contact them by phone. A pre-paid post card was enclosed with the letter; patients wishing to avoid telephone contact returned the card and were not contacted further. After a minimum of three weeks, patients not returning the opt-out postcard were contacted by telephone, screened for eligibility, and invited to participate in the study. Verbally consenting patients were mailed a study enrollment packet containing a cover letter and a written consent form. Usually within three weeks (but always prior to the next oncology visit), a study representative conducted an Enrollment Interview by phone. The purpose of the Enrollment Interview was to collect baseline information on pain, other cancer symptoms, psychological distress, health related quality of life, adherence, and self-efficacy. By collecting the majority of baseline data by telephone prior to the index visit, we limited respondent burden and were able to focus the index visit on delivery of the intervention rather than collection of data. Patients were promised a total of $80 compensation: $50 for completing the intervention and $10 for the 2-, 6-, and 12-week follow-up telephone surveys, respectively.

At the conclusion of the Enrollment Interview, patients were randomized to the TEC or EUC groups using a blocked-randomization scheme to assure balanced assignment within physicians. For each physician in the study, a separate sequence of random 1's (TEC group) and 0's (EUC group) was generated for use in assigning patients. To preserve blinding, treatment assignment (0/1) was encoded as a set of 3-digit numbers maintained by the study statistician. The encoded sequences were printed on two adhesive labels, one affixed to the patient's Enrollment Interview form and another to the Tracking Sheet in each patient's Case Report File (CRF). The CRF served as the patient's study record and included a log of all contacts with research staff, all completed telephone surveys, the survey booklet completed by the patient on the date of the intervention, and documentation of payment for study participation.

### Visit procedures

Patients were asked to arrive one hour prior to the next scheduled oncology appointment (referred to subsequently as the "index visit"). Upon arrival at the clinic waiting area, study participants were greeted by a trained Health Educator (HE), escorted to a quiet space, and given the opportunity to sign the Patient Bill of Rights, IRB-approved Consent Form, and the HIPAA Release Form. All patients then completed the Pre-visit/Pre-Intervention Questionnaire. This form was self-administered, but the HE was available to provide assistance if needed. At this juncture, patients received their assigned intervention (TEC or EUC). For quality control and training purposes, all interventions were audio-taped. Following the intervention (which lasted 20-40 minutes), patients completed another self-administered instrument, the Pre-visit/Post-Intervention Questionnaire. If the attending oncologist and patient had provided prior consent to be audiotaped (77/130 or 59% of TEC visits, 71/135 or 53% of EUC visits), the HE placed a small audiorecording device in the exam room. Immediately after the visit, patients were intercepted by the HE and asked to complete the Post-visit Questionnaire. They were thanked for their participation and reminded that they would receive their first follow-up phone call in two weeks.

### Description of experimental and control interventions

The TEC intervention was based on the model previously described by Oliver et al[13] This approach uses a specific assessment of each patient's learning needs, goals, and values to develop a set of individualized messages and skill-building exercises designed to increase self-efficacy, enhance patient-physician communication, and improve care of cancer-related pain. HEs were intensively trained over approximately 80 hours to elicit patients' values; deliver clear, plain-spoken messages; and to be sensitive to cultural differences that may influence how different patients respond to their illness and to the intervention. They were reconvened at intervals of 3-6 months to assure continued fidelity to study procedures. For this study, we revised the TEC Manual using a 6-step program: Assess, Correct, Teach, Plan, Rehearse, Portray (ACT-PReP). Components of the program include:

(1) *Assessment *of current knowledge, attitudes, and preferences (values). As an initial step in the TEC intervention, the HE reviews information supplied by the patient as part of the baseline interview, focusing on current symptoms, pain-related knowledge and attitudes, and self-efficacy expectations. Using a set of questions found in the TEC Training and Resource Manual, the HE also helps the patient to clarify his or her major pain-related concerns and treatment-preferences.

(2) *Correction *of misconceptions about cancer pain control. Patients frequently harbor false or exaggerated beliefs that can interfere with effective management of cancer-related pain[2,20,21] Using patients' responses to the Short Barriers Questionnaire,[22] the HE reviews specific pain-related misconceptions and offers an algorithm-based corrective. For example, if a patient were to believe that effective pain medicine should be withheld until the pain is so bad the patient "really needs it,", then the HE would respond that pain is easier to control when treated early, and while the dose might need to increase over time, there is no reason to hold treatment in reserve.

(3) *Teaching *of relevant concepts. Although TEC is designed primarily to enhance self-efficacy, the intervention also transmits knowledge in two domains. The first domain is *pain self-management*. Patients are taught that pain per se can be harmful to health; that pain is easier to prevent than to treat (which is why long-acting, around-the-clock, oral pain medicines are beneficial); that combinations of medicines are often required for optimal relief (e.g., short- and long-acting analgesics); that most analgesic side effects can be managed effectively (through a combination of adjuvant medications and lifestyle adjustments); and that non-pharmacologic approaches such as music, relaxation, meditation, or distraction are sometimes useful adjuncts. The second domain is *patient-physician communication*. Patients are taught that that it is important to bring pain-related symptoms to the physician's attention and that most doctors are open to a negotiated approach to care. Patients are also counseled about the possibility of physician resistance to assertive behaviors and coached regarding specific strategies for breaking through such resistance.

(4) *Planning *(identifying goals, matching strategies to goals). In this step, which is at the heart of the TEC intervention, the HE encourages the patient to identify goals, frame them so they are achievable, and plot strategies to gain the doctor's help in accomplishing them. For example, a patient might express the desire to sleep through the night without pain. The HE would ask the patient to formulate, write down, and practice questions to ask the physician what would help achieve the goal (e.g., "What pain medication can I take that will last through the night?").

(5) *Rehearsal *using role play exercises. In this step, the HE asks the patient to participate in role-playing exercises where the patient rehearses question-asking and negotiation behaviors. As a start, the educator asks the patient to practice asking the physician three (personally relevant) questions about pain. If the patient is able to complete this task successfully, the educator will ask him/her to repeat the exercise, this time introducing physician resistance (e.g., "I don't typically like to prescribe triplicate medications"; "I'd like to concentrate on getting your blood count up"; "Let's get some tests and see what's going on"). The patient will be encouraged to practice until comfortable with the behaviors or until the session ends.

(6) *Portrayal *of learned skills. In this segment, the patient has the opportunity to apply new skills immediately during the scheduled oncology visit. If the patient is successful in doing so, SCT predicts that self-efficacy will be enhanced, theoretically leading to even greater pain self-management and participation in care, which in turn leads to greater self-efficacy.[15]

Patients randomly assigned to the EUC group were greeted in the same manner and asked to complete the same survey instruments as patients assigned to the TEC intervention. The HE provided both groups of patients with a copy of the booklet, *Pain Control: A Guide for Patients with Cancer and Their Families*, published by the National Cancer Institute.[20] With EUC group patients, the HE verbally reviewed selected points in the booklet, emphasizing key aspects of pain-related knowledge, including: 1) pain is controllable with modern treatments; 2) pain medications have side effects that can be controlled; and 3) cancer patients rarely experience addiction. The main difference between TEC and the EUC interventions is that the latter promotes acquisition of knowledge in the pain domain but does not correct specific misconceptions, teach in the communication domain, facilitate planning, or encourage rehearsal of new skills. As a result, it was hypothesized that the TEC intervention would produce greater gains in self-efficacy and improved outcomes.

### Administration of measures

Table 2 describes the key measures, including constructs assessed and time points for assessment. The enrollment interview and the 2-, 6-, and 12-week follow-up interviews were conducted by phone. The Pre-visit Pre-Intervention Survey, the Pre-visit Post-Intervention Survey, and the Post-visit Survey were self-administered with assistance from the HE available as needed. *Demographic characteristics *were assessed using administrative records, the screening interview, and the enrollment interview. *Average pain *was assessed with a single numerical analog scale, with 0 representing no pain on average over the past two weeks and 10 representing the worst pain imaginable. *Worst pain *in past two weeks was assessed using the same 0 to 10 numerical analog scale. (In the pre-intervention, pre-intervention/pre-visit, and post-visit interviews, average and worst pain were measured using a visual analog scale with 0-10 numerical anchors.)*Pain impairment *was measured using the MOS Pain Impairment Scale[23]*Anxiety *was tapped by the anxiety subscale of the Hospital Anxiety and Depression Scale [24], while functional status and well-being was assessed with the SF-12.[25]*Pain-related knowledge and beliefs *were measured using 11 items selected from the Brief Pain Barriers Questionnaire, focusing on the controllability of pain, perceptions of physicians' attitudes towards pain, and concern about side effects of analgesics.[21]*Self-efficacy for controlling pain *and for *communicating about pain *with the cancer doctor were assessed based on scales developed by Anderson et al. and Maly et al. [26], respectively. *Clinical data*, including cancer diagnosis, cancer stage, current use of cytoreductive therapies, and analgesic prescribing, were obtained via chart review using a standardized abstraction form. Inter-rater reliability (kappas) for abstraction of clinical data averaged 0.94 [range: 0.84, 1.0].

**Table 2:** Description and administration of measures

Domain	Measure	Screening/Enrollment	Pre-intervention	Post-intervention, Previsit	Post-visit	2-week Follow-up	6-week Follow-up	12-week Follow-up
Demographics	Age, race, sex, education, marital status	X						

Average Pain	Numerical Analog Scale (0-10)	X	X	X	X	X	X	X

Worst Pain	Numerical Analog Scale (0-10)	X	X	X	X	X	X	X

Pain Severity	Mean of Average and Worst Pain	X	X	X	X	X	X	X

Pain Impairment	MOS Pain Impairment Scale	X	X			X	X	X

Anxiety	HADS Anxiety Subscale	X				X		X

Functional Status and Well-being	SF-12	X					X	

Depression	PHQ-2 (not administered until Spring 2007)	X				X	X	X

Pain Beliefs	Brief Pain Barriers Questionnaire		X			X		

Pain-Related Self-Efficacy	Derived from Anderson	X	X	X	X	X	X	X

General Adherence	MOS General Adherence	X				X	X	

Communication Self-Efficacy	Maly	X	X	X	X	X	X	X

Analgesic Therapy	Chart review				X			

For the subsample of visits that were audiorecorded (n = 148), two sets of communication measures were used, discourse coding of active patient participation and observer ratings of physician's informativeness and facilitative communication [27-30] Using a previously validated coding scheme,[28,30] active patient participation was coded independently by two coders who identified three categories of utterances: asking questions, assertiveness (offering opinions, stating preferences, making a request), and expressing concerns (worries, fears, negative feelings). The intraclass reliability coefficient (ICC) for the sum of active communication behaviors, established on subset of 15 consultations, was 0.78. Two coders also listened to each interaction and rated the physicians' informativeness on 5-point Likert scales[27,29] and the physicians' participatory decision-making on 10 point scales[31,32] The ICC was 0.80 for informativeness and 0.72 for participatory decision making.

### Sample size determination

A prospective sample size calculation was performed during the protocol-writing stage. It aimed to determine a target sample size that would provide 80% power for two-tailed testing (at a type-1 error rate of 5%) of each of the key study hypotheses concerned with between arm differences in regression-adjusted follow-up outcome measures. The investigators concluded that an effect size of 0.33 units of standard deviation of the unadjusted outcome distribution was appropriately modest for a relatively inexpensive pain control intervention. Under plausible assumptions about the net variance reduction achievable through particular features of our study design (e.g. within-physician randomization and repeated measures regression data analysis)[33] and about the rate of attrition for enrolled participants after study baseline, a sample size of 230 was targeted as being sufficient to achieve the required effective sample size of 284. A power analysis conducted independently by the Data Safety and Monitoring Board early in the data collection phase of the study resulted in the recommendation that the target sample size be increased to 275. The targeted sample size of 275 provides the desired statistical power under more conservative assumptions about the net effects of sample attrition (up to 15% of patients lost to follow-up at 6 weeks), variance reduction via regression adjustment (no more than 25%) and variance inflation of up to 10% arising from between-physician heterogeneity (e.g. clustering effects) in treatment effects.

### Data Safety and Monitoring Board (DSMB)

The first patient was enrolled on 10/30/2006. A DSMB was established shortly after project launch and met several times during the two-year data collection period. Members included a clinician-epidemiologist, an attorney affiliated with a non-profit medical research and policy foundation, and a senior academic statistician who served as the Board's chair. Based on the Board's recommendations issued in June 2007, project staff amended the study protocol to limit the number of enrolled patients per physician to a maximum of 40; increase the target sample size from 230 to 275; and develop procedures for dealing with (and if necessary, referring) patients who were disturbed by the screening phone call because they were no longer in active treatment for their cancer. In the subsequent project year, the Board evaluated randomization procedures and requested additional analyses following the revelation that among the first 100 enrollees, intervention group patients were significantly older than control group patients. The Board found no irregularities and attributed the age imbalance to chance.

### Patient accrual and study flow

**Figure 3:**
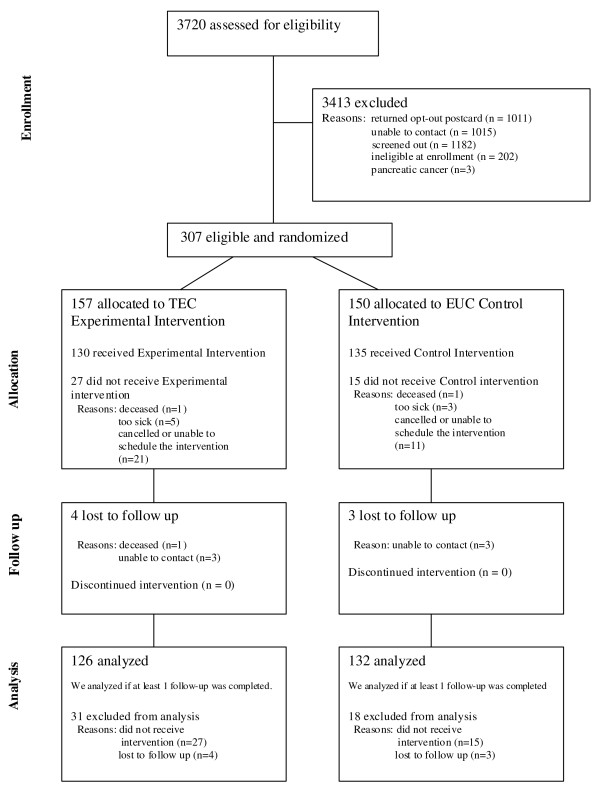
**Flow of subjects in trial**.

Of 3720 patients sent a recruitment letter, 3413 were excluded due to returning an opt-out postcard (n = 1011); inability to contact by phone despite repeated calls (n = 1015); or ineligility at the time of screening (n = 1182), at enrollment (n = 202) or during follow-up (i.e., 3 patients with pancreatic cancer were dropped from the study on the advice of the DSMB) (Figure 3). The remaining patients (n = 307) were randomized, and 265 received the allocated treatment (130 received the experimental [TEC] intervention, 135 received the control intervention).

### Planned analytic approach

In subsequent publications, we will report results pertaining to intervention effects and putative mechanisms. Primary outcomes will include: 1) pain severity at two-weeks, measured as the mean of average and worst pain and 2) pain-related impairment at 2 weeks. To test for attenuation, the effect of the intervention on pain severity and impairment at latter follow-up occasions (i.e. at 6 and 12 weeks) will be reported as secondary outcomes. Additional secondary analyses will estimate effects of the intervention on average pain; self-efficacy for pain control; and self-efficacy for communication with the physician. Finally, exploratory analyses will assess the interaction between the intervention and minority race, as suggested by previous work[14] The primary estimates of the effects of the intervention on each of these outcomes will come from generalized linear regression analyses, following the intent-to-treat principle. All analyses will adjust for the nesting of observations within physician, and, where appropriate, the nesting of repeated observations within patient, with nesting variables treated as random effects.

## Discussion

The Ca-HELP Study is a multi-site randomized trial of a patient-centered coaching intervention designed to improve cancer pain care and outcomes. Important features of the study include its focus on ambulatory patients with cancer-related pain recruited from several health care systems, broad inclusion criteria, strong theoretical foundations based on SCT, randomization at the patient level, and tracking of multiple outcomes at 2, 6, and 12 weeks. Use of multiple methods (survey, audiorecording) and multiple measures (pain, quality of life, self-efficacy, communication) favors a comprehensive assessment of a complex behavioral intervention. We have demonstrated the ability to train lay HEs with high fidelity and recruit, enroll, and track large numbers of patients with cancer-related pain.

Major challenges encountered during implementation of the study included recruiting and training HEs, complying with multiple Institutional Review Boards (IRBs), maintaining fidelity of the intervention, and dealing with patient fatigue and distress during advanced stages of illness. Seeking generalizability, we initially recruited a relatively large cohort of part-time lay HEs but found turnover was reduced by relying on a smaller number of more committed personnel. Recruitment of patients was slowed by IRB requirements that patients identified as appropriate candidates by their oncologists be allowed the opportunity to opt out by mail before being contacted by the study staff. However, these requirements were an artefact of the study and would not apply should the intervention be incorporated into routine clinical care. Fidelity to the ACT-PReP paradigm was ensured by universal monitoring and intermittent review and feedback. Finally, while the vast majority of patients expressed gratitude for being able to participate in the study, some had trouble with lengthy telephone follow-up instruments, which occasionally required interruption of the interviews with call-back at a more convenient time.

In summary, the Ca-HELP Study has successfully trained lay HEs to deliver a theory-driven intervention designed to improve an important clinical target. Subsequent analyses will focus on the effects of the experimental intervention on pain, self-efficacy, and quality of life and examine relationships among processes and outcomes of cancer pain care.

## Competing interests

The authors declare that they have no competing interests.

## Authors' contributions

RLK generated the idea, obtained funding, and orchestrated implementation of the protocol. DJT contributed to the design and statistical analysis plan. RLS planned the clinical conversation analysis. DK contributed specialty expertise in pain management. TG coordinated implementation of the study at the HMO site. TW coordinated implementation at the academic site. CS and DED managed the project. LL served as lead health educator. PF contributed to all aspects of design and analytic planning. All authors read and approved the final version of the manuscript.

## Pre-publication history

The pre-publication history for this paper can be accessed here:

http://www.biomedcentral.com/1471-2407/9/319/prepub
